# Healthcare professionals’ views of the use and administration of two salvage therapy drugs for acute ulcerative colitis: a nested qualitative study within the CONSTRUCT trial

**DOI:** 10.1136/bmjopen-2016-014512

**Published:** 2017-02-20

**Authors:** Clare Clement, Frances Rapport, Anne Seagrove, Laith Alrubaiy, John Williams

**Affiliations:** 1 School of Social and Community Medicine, University of Bristol, Bristol, UK; 2 Centre for Healthcare Resilience and Implementation Science, Macquarie University, Sydney, Australia; 3 Swansea University Medical School, Swansea University, Swansea, UK

**Keywords:** QUALITATIVE RESEARCH, health professionals, ulcerative colitis, infliximab, ciclosporin

## Abstract

**Objectives:**

Insight into healthcare professionals’ views and experiences of the use of ciclosporin and infliximab as salvage therapies for acute ulcerative colitis (UC) and how this may affect participation in a comparison trial is lacking. The study aimed to capture views and opinions of healthcare professionals about the two drugs within the CONSTRUCT trial.

**Design:**

An interview-based qualitative study using Framework Analysis embedded within an open-label, pragmatic randomised trial.

**Setting:**

National Health Service Health Boards and Trusts, including large teaching and district hospitals in England, Scotland and Wales.

**Participants:**

Principal Investigators (PIs) for trial sites (who were all consultant gastroenterologists) and nurses responsible for administering and monitoring the salvage therapy drugs across trial sites. 15 PIs and 8 nurses recruited from a range of sites stratified by site recruitment rates were interviewed.

**Results:**

Interviews revealed that professionals made judgements regarding the salvage therapies largely based on experience of giving the two drugs and perceptions of effectiveness and adverse side effects. A clear preference for infliximab among nurses was revealed, largely based on experiences of administration and drug handling, with some doctors strongly favouring infliximab based on experience of prescribing the drug as well as patient views and the existing evidence base. Most doctors were more equivocal, and all were prepared to suspend preferences and wait for evidence of effectiveness and safety from the CONSTRUCT trial. PIs also questioned guidelines around drug use and restrictions placed on personal autonomy in delivering best patient care.

**Conclusions:**

Findings highlight healthcare professionals’ preference for the salvage treatment, infliximab in treating steroid-resistant UC, largely based on resource intensive nursing requirements of intravenous administration of ciclosporin. Not all doctors expressed this preference, being more equivocal, and all professionals were content to suspend preferences within the CONSTRUCT trial and recognised the importance of establishing relative effectiveness and safety.

**Trial registration number:**

ISRCTN 22663589.

**Strengths and limitations of this study**

- The first study that has successfully incorporated qualitative data into a clinical trial to explore professional equipoise, preference and drug management for salvage therapy in patients with ulcerative colitis who fail to respond to steroids.
- The study cannot make any claims about the views of the full professional trial cohort but data collected can reveal current trends in practice and professional opinion to inform service development and future drug use.
- Interview data, as an integral part of trial findings, provided insight into factors influencing trial treatment preferences and addressed issues of equipoise within the trial.

## Background

Ulcerative colitis (UC) is a chronic debilitating disease which affects ∼150 000 people in the UK.^1^ In many cases, UC presents as acute severe colitis requiring patient hospitalisation;^2^ however, there are unanswered questions regarding causes, course, treatments and outcomes, despite rapidly developing new therapies. The standard approach to treatment is administration of intravenous steroids, but about 40% of patients fail to respond to this.^3^ ^4^ Until recently, emergency colectomy remained the only option when steroids fail, with a high mortality rate.^5^ While there are now a range of other treatments, and mortality following colectomy has dramatically reduced, 10% of patients still die within 3 months of surgery.^5^

Infliximab and ciclosporin are immunosuppressant drugs that offer hope for the treatment of steroid-resistant UC. There is evidence that both are effective in the shorter term,^6^ ^7^ but questions remain over their longer term clinical use and cost-effectiveness and little is known of the views and experiences of patients and clinicians regarding the two drugs.

The ‘Comparison Of iNfliximab and ciclosporin in STeroid resistant Ulcerative Colitis: a Trial’ (The CONSTRUCT trial) aimed to compare the clinical and cost-effectiveness of infliximab and ciclosporin for patients with steroid-resistant UC using quantitative, qualitative, health economic and data-linkage research methods^8^ ^9^ in a multicentre UK-wide pragmatic randomised controlled trial. Following explanation and consent, 1614 patients who were admitted with acute severe UC were recruited into a cohort from which 270 who failed to respond to intravenous steroids were recruited into the trial. The trial recruited patients from 52 hospitals between May 2010 and February 2013, and investigated differences between the two drug treatments in terms of: patient health-related quality of life, adverse events, mortality, disease activity, colectomy rates and cost-effectiveness. Additionally, the trial aimed to enrich trial findings and reporting of treatment delivery context by seeking the views and experiences of patients about treatments and changes to health over time, and the opinions of healthcare professionals about drug outcomes, ease of drug handling and drug preference. The CONSTRUCT Trial Management Team was also aware that potential treatment preferences within trial healthcare professionals could influence recruitment to the trial^10^ and therefore sought to also explore this within healthcare professional interviews.

It is important to understand context of delivery, implementation, perceived outcomes and treatment preference in order to fully understand and report pragmatic trials.^10^ ^11^ Through understanding how the investigative drugs are managed and perceived, trial findings become more patient centred and sensitive to the context of practice. What little insightful experience-based research is reported in the literature on ciclosporin or infliximab focuses on infliximab's role in treating rheumatoid arthritis.^12^ There are no studies exploring the use of ciclosporin in the treatment of acute UC and what people's experiences and perceptions are of this treatment. One qualitative study exploring patient and parent experiences of infliximab in gastroenterology paediatric settings found favourable views of the drug when used in a hospital environment.^12^ However, to the best of the authors’ knowledge, no studies, qualitative or otherwise, have explored health professionals’ views of these drugs in terms of their administration, ease of handling or value for treating steroid-resistant UC. This is somewhat surprising, in view of the fact that qualitative methods are particularly well suited to the investigation of personal experience, individual perception and belief and meaning systems^13^ ^14^ and can enable researchers to clarify patients’ and clinicians’ understandings of clinical practice and drug regimes. This is particular pertinent to trials where such views and experiences can influence trial conduct.^15^ ^16^

In the light of the lack of current understanding and an awareness of the importance of drug preference, delivery context and drug management practices within the CONSTRUCT trial, it was deemed vital to seek both patients and healthcare professionals’ opinions. This paper focuses solely on the professional views, while patient views and experiences are presented elsewhere.^9^

### Study aims and objectives

The study aimed to gain insight into the views and opinions of healthcare professionals about the drugs being tested in the CONSTRUCT trial (ie, infliximab and ciclosporin) and their personal contribution to the trial. Specific objectives were to examine how drugs were administered, their perceived ease of handling, professionals’ personal drug preferences and views on colleagues’ drug preferences, and equipoise in recruiting to the trial.

## Methods

An interview-based qualitative study was embedded within an open-label, pragmatic randomised trial.

### Participants

Principal Investigators (PIs) for trial sites (who were all consultant gastroenterologists) and nurses responsible for administering and monitoring the drugs across trial sites were included in the interview cohort. Multidisciplinary team views would offer insights into drug administration and personal preferences, and clarify professionals’ opinions of the physical effects of both drugs on patients and trial participation. A purposive sampling method was employed to select PIs for interview, based on recruitment performance of sites: recruiting well to cohort and trial; recruiting well to cohort but less well to trial; and those recruiting poorly to both the cohort and the trial. Nurses were only recruited from sites recruiting well to cohort and trial, in recognition of the fact that other nurses would have had little relevant experience with one or other of the two trial drugs.

### Interviews

The PIs and nurses were approached via email or phone, and were provided with information about the interview study including aims and topics to be discussed during the interview. Participants gave informed consent through email responses. Experienced qualitative researchers (CC and AS) used a semistructured interview approach, allowing interviewees to expand on responses to questions and raise issues of importance.^14^ CC was not known to the participants before interviews but AS had previous contact with them within the trial. The interview schedule covered: interviewees’ beliefs and ways of working; aspects of drug provision that affected preferences, ease of drug handling, effects of drugs; patient interaction; professional communication; views on other professionals’ response to drug provision, and personal contributions to the trial, including issues of equipoise (interview topic guide is available as online supplementary material). Interviews were carried out face to face or over the phone, and lasted between 30 and 40 min. PIs and nurses were interviewed separately to ensure that an understanding could be gained of differences between them. Interviews were audio recorded and transcribed for analysis purposes. A pilot PI interview was conducted to test the schedule and process. Piloting resulted in no changes.

10.1136/annrheumdis-2016-210131.supp1 supplementary material

### Analysis

Transcripts were analysed using ‘Framework Analysis’^17^ to develop a thematic template that linked data to study aims and objectives and guide analysis. Framework analysis is a semistructured approach that, like generic thematic analysis, uses coding to conceptualise and classify research data.^18^ Three qualitative researchers (CC, AS and FR) individually coded transcripts as they were transcribed, increasing analysts’ knowledge in an iterative fashion as each new transcript was received and transcript data supporting the analysis framework were added. The three then worked together to derive a coding structure including essential and incidental themes and their linked categories within the framework. Continuing group work to refine the framework validated the process and ensured that major themes retained their nuance and detail. The analysts monitored the process throughout data collection and analysis until data saturation (when no new information was revealed to further theme development or understanding) was achieved.^19^ ^20^ Data saturation was achieved following the completion of 23 interviews as agreed by the research team.

## Findings

The interview cohort comprised 15 PIs (consultants) and 8 nurses. The PI stratification was as follows: eight from sites recruiting well; four from sites recruiting well to cohort but less well to trial and three from sites recruiting poorly to both cohort and trial. The nurses were all from high trial recruitment sites (the same sites as the 8 PIs). Not all PIs agreed to participate, due to busy work schedules. Those declining were from lower recruiting trial sites.

Through the Framework Analysis, four main themes emerged: (1) drug administration and management, (2) personal preference and trial involvement, (3) policy development and drug regulation and (4) patient benefit and negotiated care. Both PIs and nurses discussed all four themes to an extent with overlapping views and experiences; however, it should be noted that nurses’ views were largely based on experiences of administration and drug handling. PIs views were largely based on experience of prescribing the drug as well as patient views and existing evidence base. Themes were underpinned by a rich data set comprising: (1) descriptions of the practicalities of administering and monitoring the two drugs and (2) contextual factors influencing professionals’ views of the effects of infliximab and ciclosporin on the alleviation of symptoms and on patients’ quality of life. In addition, analysis helped derive: (3) greater understanding of professional personal preference for one or other drug, (4) knowledge of professional experience and entry into the trial, (5) information about other healthcare professionals’ practices and (6) perspectives on current Government policy regarding the regulation of drugs and views on National Institute for Health and Care Excellence (NICE) Guidelines.^21^ Underpinning all of this was an ongoing concern for patients’ welfare, informed patient choice and joint decision-making.

The main findings are described in detail below alongside professionals’ verbatim quotations. All quotations are numbered, refer to table 1, coded as ‘PI’ or ‘nurse’ and include indication of which trial recruitment cohort.

**Table 1:** Quotations

Main theme	Quote number	Quote
Drug administration and management	Q1	It [ciclosporin] goes on over a longer period of time obviously, so the need [for nurses] to continually make up bags over a longer period of time rather than just the one off infusion. (PI) Recruiting well
	Q2	It's [ciclosporin] time consuming for the nurses and slightly messy…it's complicated and it's work for the nursing staff. (PI) Recruiting well
	Q3	Having to make it up [ciclosporin] every 6 hours is time consuming, changing lines, always having two nurses to check it because the way the GI unit is split…there's a corridor between the two wards so obviously bed cover etc but only to have one trained [nurse] each side is a bit difficult … geography of the wards … we've no one else to check the drugs. (Nurse) Recruiting well
	Q4	[Ciclosporin] is fairly cumbersome for both the staff and equally importantly for patients because once you are tied to the drip and associated drip stand, it restricts patients moving around. (PI) Recruiting well
	Q5	Infliximab has an advantage (over ciclosporin) in that it's just a two-hour infusion and then it's done… there's no problem with infliximab, I think it's a good drug to administer, I think it's an easy drug to administer… I like infliximab because once you've done it, you've done it for 2 weeks. (PI) Recruiting well
	Q6	Tend to use ciclosporin as acting more quickly. (PI) Recruiting well to cohort but less well to RCT
	Q7	This is the one thing I don't like about infliximab, because there is no data to give us a clear timescale or timeline for decision-making. (PI) Recruiting well
	Q8	I'm always a little bit more nervous with it [ciclosporin] … and I think that's from the side effects of renal impairment … I think the side effects profile of ciclosporin, still concerns us more. (PI) Recruiting well
	Q9	Potentially quite significant side effects… slightly uncomfortable feeling about it [ciclosporin]. (PI) Recruiting well
	Q10	It's [ciclosporin] time consuming with regards to observation, particularly on a busy ward when you've got one nurse to 10 patients, it can take quite a huge part of your workload. (Nurse) Recruiting well
	Q11	We have to monitor this patient closely for any side effects … it [ciclosporin] takes three hours because you've got to do obs [observations] for a couple of hours, but then sometimes we tend to forget because we get busy with other patients … so ideally it should be one to one. (Nurse) Recruiting well
	Q12	Most of the time it [infliximab] goes without incident. (Nurse) Recruiting well
	Q13	Have only ever seen minor reactions to the infliximab. (Nurse) Recruiting well
	Q14	We now have to request the funding for patients with UC on long-term infliximab so that's a bit of a challenge and it means that decisions are made differently to whether a patient is going onto maintenance infliximab. (Nurse) Recruiting well
	Q15	It's already an issue because we can't really treat as maintenance with infliximab so obviously if a patient responds well we've had to get exceptional funding and things like that so there is a case for it I think. (Nurse) Recruiting well
Personal preference and trial involvement	Q16	My personal view is that they probably have equal efficacy, I think infliximab has less side effects so if I was given a free choice, if I was asking for me or for my loved ones, I would opt for infliximab. (PI) Recruiting well
	Q17	When a patient was given infliximab I was rather pleased and when they were given ciclosporin I was less enthusiastic … we wondered about the tolerance for the patient and convenience for the patient. (PI) Recruiting well
	Q18	I prefer it when they have infliximab … I think it's easier for the patients because it's just one infusion…it's a couple of hours, instead of being hooked up. I mean keep in mind they have profuse diarrhoea… I think it's easier to administer as a nurse and it's easier for the patient to receive. (Nurse) Recruiting well
	Q19	Rather more relaxed about it [ciclosporin], paradoxically, simply because I know when the IV infusion is stopped then the drug disappears. (PI) Recruiting well
	Q20	I've used it [ciclosporin] for a very long time and I'm comfortable with it. (PI) Recruiting well
	Q21	If the result was that ciclosporin was a lot better we'd use it and if the results were that infliximab was a lot better we'd use it. (PI) Recruiting well
	Q22	The difference (between drugs) is not sufficiently dramatic that I would say it was unethical to put them into a randomised study. (PI) Recruiting well
Policy development and drug regulation	Q23	There is an issue of cost of course, [infliximab] we're pushed all the time to stop it for cost reasons. (PI) Recruiting well
	Q24	It's very hard to find anyone who supports the NICE guidance in its present format. (PI) Recruiting well
	Q25	I sort of treat it with a bit of contempt. (PI) Recruiting well
	Q26	You can usually find a reason why ciclosporin would be contraindicated…I don't think it's a particularly sensible NICE guidance based on the lack of evidence that they have to make their decision and therefore in all honesty because we can, we slightly ignore it. (PI) Recruiting well
Patient benefit and negotiated care	Q27	I like to tailor the treatment to the patient…to the patient's previous history, patient's wishes (PI) Recruiting well to cohort but less well to trial
	Q28	The drug we choose ultimately depends on the patient. (PI) Poor overall recruitment
	Q29	It's whatever is best for the patient … I think it's different for each patient and I think that they need to be given the choice. (Nurse) Recruiting well
	Q30	Our surgeons are very active in decision-making, and have multi-disciplinary team meetings twice a week. (PI) Low overall recruitment
	Q31	Care is negotiated with the patient, patient input is important, but pre-defined pathways are according to previous discussions. (PI) Recruiting well to cohort but less well to trial
	Q32	Patient views are as important as anything else, combined with weighing up risks and benefits, we involve them hugely. (PI) Recruiting well to cohort but less well to trial

### Drug administration and management

PIs and nurses, when asked about the administration of infliximab and ciclosporin, were keen to point out that consideration must be given to the administration process as well as to the lead-up procedure: ‘workup to treatment’ (PI, Recruiting well to cohort but less well to trial). Patient workup was seen as a vitally important step in administering the drugs. However, workup was said to be something that was neither given enough consideration within the drug guidelines, nor discussed in terms of its effect on the rest of the administration process, such as its effect on drug handling. Interviewees described the workup as necessary for the provision of infliximab and ciclosporin in similar terms; however, following initial workup, administration of infliximab was much easier to handle than the administration activities necessary for the provision of ciclosporin. First, there was less to be concerned about during that period of time and second, the process had less impact on workload, especially for nurses, while both drugs demanded due care and attention. Attention to detail was described according to: the careful mixing of drugs and other preparations necessary for treatment, prescreening of patients and patient preparedness for the intravenous infusions. In this respect, ciclosporin was consistently described as the more complicated of the two, with a longer administration time (continuous infusion as opposed to 2 hours for infliximab) and with the need for frequent changes of intravenous bags (infliximab is a one-off infusion). This had a knock-on effect on healthcare professionals’ work schedules: see Q1 and Q2.

Nurses’ views accorded with PI opinion on this matter. In addition, nurses were keen to emphasise the complexities resulting from contextual and geographical factors that had to be contended within a busy hospital setting, such as managing patients on different wards or requiring an infusion, and as a consequence the need for a greater number of nurses with advanced prescribing capabilities on the wards at any one time: see Q3.

The practicalities of a lengthier ciclosporin infusion had implications for patients’ well-being too, with health professionals having to spend more extensive periods of time in hospital dealing with patients who were: ‘frustrated’ (Nurse, recruiting well) with highly restricted movement: see Q4.

Views on the ease of administering individual drugs were clearly influenced by an individual's familiarity with the drug in question. Thus, those with more experience of ciclosporin tended to be more positive about that drug, and they suggested that nurses should be trained in both drugs as they faced a steep ‘learning curve’ (PI, recruiting well), particularly in relation to ciclosporin. Nevertheless, support for the administration of infliximab was more strongly felt and it was repeatedly described as the ‘easier’ (PI, recruiting well) and more ‘convenient’ (PI, recruiting well) drug: see Q5.

While both drugs were perceived to be effective, ciclosporin was faster-acting, and offered a clearer patient response timeline. Infliximab, on the other hand, presented a challenge in terms of whether to continue with it or look for a different treatment. Ciclosporin was more ‘clear-cut’ (PI, recruiting well) and PIs felt more confident in their decision-making and timing around prescribing this drug. In addition, PIs worried about the limited information available for infliximab response rates, as this did not appear to match patients’ actual responsiveness, made all the more complex by the need for an extended administration period: see Q6 and Q7.

In addition to response times, adverse effects were of major importance in treating patients with one drug or another. Ciclosporin, in particular, was seen to have a range of associated adverse effects, which included: drug toxicity, renal failure, neurological impairment, seizures, tremors and hypertension. PIs were ‘uneasy’ (PI, recruiting well) about the drug, seeing it as ‘dangerous’ (PI) poor overall recuitment. Even those advocating its use tended to be apprehensive about the adverse effects in the longer term, viewing it as more of a bridging therapy. These views were reinforced by a perceived lack of evidence of ciclosporin's longer term benefits. See Q8 and Q9.

An adverse risk profile for ciclosporin was described as resulting in increased patient monitoring and checking of drug and blood levels, which, in addition to a more resource-intensive process, impacted on nurses’ time. This is particularly noticeable on a busy ward where there are difficulties with nurse–patient ratios, and nurses had the sense that their profession was struggling with workload increases. This impacted on their ability to give equal time to all patients under their care. They reported occasionally: ‘forgetting’ (Nurse, recruiting well) to monitor patients on ciclosporin. Infliximab, on the other hand, did not warrant any additional monitoring following inpatient discharge and no issues associated with monitoring were raised: see Q10 and Q11.

While infliximab also carried a risk profile that included adverse reactions, causing particular hesitancy in prescribing it for the older patient, it was favoured for its perceived fewer side effects and longerterm efficacy: see Q12 and Q13.

However, PIs pointed their frustrations with policy restrictions and Government guidelines that prevented the use of infliximab in the way they wished, including the need to justify its use in the longer term: see Q14 and Q15.

### Personal preference and trial involvement

PIs clearly recognised the effect that their own experience of using one drug or another had on their personal preference. While many were equivocal, some PIs expressed a clear preference for infliximab over ciclosporin based on its ease of preparation, easier administration, lower negative impact on nurse care provision, staffing and workloads, longer term benefits and reduced adverse effects. In addition, both PIs and nurses pointed to greater patient tolerance and enhanced patient convenience. Further reasons for stated preferences included: familiarity with the drug, drug effectiveness, greater clinical expertise and knowledge, and a general sense of ‘unease’ (PI, recruiting well) with the use of ciclosporin: see Q16 and Q17.

Nurses’ views were similar to those of their consultant counterparts, expressing a preference for infliximab based on its ease of administration, perceived patient benefits and personal experience and familiarity with the drug. They highlighted the restrictions of patients being hooked up to a ciclosporin infusion for longer periods of time while suffering from bowel disturbances, and noted patients’ preference for infliximab in this respect. Ciclosporin was described as ‘high maintenance’ (Nurse, recruiting well): see Q18.

Two PIs described a preference for ciclosporin, based on personal familiarity with the drug, its quicker-acting properties and its ability to disappear from the system once the drug administration had been halted. However, even these two interviewees stressed the need to retain a level of vigilance—to ‘be more wary’ (PI, poor overall recruitment): see Q19 and Q20.

In spite of these personal preferences, there remained a genuine sense of uncertainty as to which drug was the more beneficial at the time of the trial, particularly in relation to people's views of trial entry, trial management and patient recruitment. Most healthcare professionals commented that the relative effectiveness of the two drugs was unclear, genuinely wanting answers from the trial team as to the ‘better’ (PI, recruiting well) of the two. This desire was described as patient-driven, based on which of the two was most effective and well received by patients: see Q21 and Q22. All professionals expressed contentment to suspend preferences within the CONSTRUCT Trial and recognised the importance of establishing relative effectiveness and safety.

Professionals welcomed the CONSTRUCT trial, and congratulated the trial team for moving this body of work forward so successfully. They discussed the fact that they had attempted to actively recruit patients into the trial, despite, in the lower recruiting sites, a lack of success, which was put down to busy workloads and lack of joined-up working patterns.

### Policy development and drug regulation

Healthcare professionals were clearly aware of the higher cost of infliximab, and saw implications of this driving policy development and guidelines for practice. Guidelines around the use of the drug ultimately affected clinical decision-making: see Q23.

While healthcare professionals understood the need for rigorous guidelines around the use of the drugs, they also linked guidelines to an enforced restriction of the use of infliximab, arguing that the NICE guidelines (referring to TA140)^21^ were outdated, outmoded and out of step with more recent evidence. This left them, as a body of healthcare professionals, feeling frustrated and constrained in their ability to provide the best and most appropriate care for their patients. They described having ‘their hands tied’ (PI, poor overall recruitment), and believed that the more recent clinical evidence and their own personal experience indicated infliximab as the drug that needed to be used more widely. As a consequence, they urged that guidelines become more flexible in order to accommodate patient needs more appropriately: see Q24 and Q25.

The point was repeatedly reinforced. While the guidelines were there to be adhered to, there was a clear sense of rebellion within the profession, with clinicians ‘ignoring’ (PI, recruiting well) guidelines to meet patient needs, in order to administer infliximab. Thus healthcare professionals welcomed a much-needed change to the guidelines that allowed for greater flexibility of drug use, led primarily from an assessment of patient need: see Q26.

### Patient benefit and negotiated care

Underpinning discussions around the administration of the drugs, treatment of patients, personal preference, and adherence to guidelines and regulations was a professional focus on patient benefit and need. Nurses and PIs gave many examples of the decisions that took account of what was best for the patient, especially when it came to a consideration of the patient's personal circumstances: see Q27 and Q28.

Nurses appeared more influenced than PIs by the patient experience, perhaps as a result of their extended contact with the patients, indicated by the fact that nurses always discussed their preference in the context of patient convenience and quality of life: see Q29.

As evident in quote 29, the notion of negotiated care was central to the consultation as far as nurses were concerned and ongoing patient care, and decision-making was perceived as a shared practice across and within multidisciplinary teams. This led to a shared professional responsibility, while the patient–clinician interaction came across as the ultimate example of this: see Q30, Q31 and Q32.

## Discussion

Professional interviews revealed that PIs and nurses make judgements regarding the salvage therapies largely based on experience of giving the two drugs and perceptions of effectiveness and adverse side effects. Professionals also recognised restrictions placed on patients having continuous infusion of ciclosporin and the ensuing frustration. Within this context, doctors tended to favour infliximab, wishing to see it as the drug of choice in view of its ability to deal with the many complex symptoms this disease group displays, fewer side effects, greater familiarity, greater effectiveness and ease of handling. Such factors have been recognised as influencing doctors’ prescribing habits for new drugs in the existing literature^22^ and clearly influenced which drug professionals prefer within this study.

Ciclosporin was presented as more complex and cumbersome to deliver, which required additional monitoring which tended to be particularly problematic on busy wards with extensive demands on an already overstretched healthcare professional workforce. This led to nurses presenting a clear preference for infliximab. Furthermore, ciclosporin was considered excessively demanding of specialist nurses’ and other healthcare professionals’ time, who need to be present to manage any complications and the drug's adverse risk profile required a greater perceived need for more intensive patient monitoring. This is a matter of concern in the light of contemporary reports regarding patient care and safety implications of overworked and understaffed hospitals and is contrary to recommendations to increase the support provided by non-medical professionals.^23^

Consequently, professionals questioned the current NICE guidelines (TA140)^21^ and government regulations around drug use, and PIs presented a sense of unease about the restrictions placed on their personal autonomy in prescribing infliximab to patients with acute UC who failed to respond to conventional therapies. Professionals were keen to change the regulations surrounding current prescribing and drug administration, and they also wanted changes to be implemented regarding longer term patient maintenance, with many preferring infliximab as the longer term drug of choice.

The current literature on inflammatory bowel disease (IBD) and associated diseases^24^ ^25^ supports this view, describing the disease group as displaying particularly complex activity and requiring a range of tools and treatment algorithms to assess symptoms and treat appropriately. Consequently, these professionals were keen to understand the individual patient's perspective as to how the disease affects their life. The literature suggests that such an understanding is closely aligned to the patient's health and social care needs and therefore should not be overlooked^25^ ^26^ To take account of individual perspectives, professionals recommended good communication, including shared decision-making over symptom relief and drug prescribing. The literature endorses this finding, highlighting the value of close empathic professional support for patients with IBD and other related diseases^27^ and emphasises the extensive psychosocial concomitants of this illness group, including a positive association with poorer perception of health and well-being and poorer psychological function.^28^

### Strengths and limitations

The results of this qualitative interview study indicate the importance of incorporating qualitative data within clinical trials and how a deeper understanding of health professionals’ views can clarify clinical data about drug treatment and drug preference. This includes how preferences can affect or, in this case, not affect equipoise and participation in the trial. Interviews with PIs and nurses who were treating participants in a large UK-wide randomised controlled trial to collect rich, complementary and comprehensive data contributed to the study's aims and objectives. Qualitative data came from interviewees in a broad mix of participating sites (recruiting well to cohort and trial, recruiting well to cohort but less well to trial and those recruiting poorly to both the cohort and trial). The data collected provided an understanding of healthcare professionals’ perspectives on patient recruitment and the two drugs. Interviews have illuminated valuable professional perspectives, filled gaps in our knowledge base about personal views and experiences of the two salvage drugs within UC and provided joined-up trial outputs and ideas for follow-on qualitative research with further trial professionals.

This study has its limitations; whereas qualitative research does not seek population representativeness or provide generalisable findings, the small interview numbers could be considered a limitation and without an extension to the sample size, to include further healthcare professionals who recruited for the cohort and the trial, we cannot make claims for the overall views of the professional trial group. Nor can our claims about patient recruitment, trial success, shared decision-making and drug equipoise be transferable across all trial sites. For this, further interviews are needed to usefully build on these data to take findings forward. Future research should focus on exploring findings from this study across a greater number and wide range of trial sites. It would also be beneficial to explore, following publication of the main trial findings, how views may have changed in the light of equal effectiveness across the two trial drugs being shown.^29^ How will this influence the preferences stated here and will it affect professionals’ decision-making between the two?

## Conclusion

To conclude, this paper has highlighted strong healthcare professional support for infliximab as salvage treatment for patients with acute severe UC who fail to respond to steroids, largely based on the resource-intensive nursing requirements for the intravenous administration of ciclosporin. However, in spite of drug preferences in some, professionals were happy to suspend any such preferences while participating in the trial and recognised the importance of establishing relative effectiveness and safety. It is apparent that professionals were influenced by their own personal familiarity with the drug and considerations of its perceived effectiveness and benefit for patients. They are also swayed by their views of others’ ability to manage drugs, and their effectiveness in the longer term for patient health and well-being. Nevertheless, professionals are keen to seek answers to questions from the trial as to which is the more beneficial of the two drugs, seeing the trial as a vital step towards providing the information necessary to take practice forward. For the time being, at least, professionals will continue to uphold their own practices, circumventing the rules and regulations to some extent, in order to offer patients their drug of choice.
