# Supplementary material for: Healthcare professionals’ views of the use and administration of two salvage therapy drugs for acute ulcerative colitis: a nested qualitative study within the CONSTRUCT trial
Source: BMJ Open. 2017 Feb 20;7(2):e014512. doi: 10.1136/bmjopen-2016-014512 (PMC5337666; doi:10.1136/bmjopen-2016-014512)
Supplement: supplementary material [file bmjopen-2016-014512supp.pdf]

Healthcare professionals' views of the use and administration of two salvage therapy drugs for acute ulcerative colitis: A nested qualitative study within the CONSTRUCT trial

Supplementary Information

Interview Topics

Administration of the two drugs

Views on IV ciclosporin

Views on infliximab

Cost implications of the drugs and administration

Treating patients with the two drugs

Treating a patient with IV infliximab

Treating a patient with ciclosporin

Infliximab in the longer term

Ciclosporin in the longer term

Advantages and disadvantages of use, administration and drug handling

Drug benefits

Patient-centred care approaches

Interaction with patients and other healthcare professionals

Personal preferences and views of colleagues' preferences

Nurses' views on doctors' preferences

PIs' views on nurses' preferences

Surgery

Colectomy as a treatment as opposed to medical intervention

National Institute for Clinical Excellent (NICE) Guidelines [refers to TA140, April 2008]

NICE guidelines on infliximab use in acute UC

Views on the Trial

Equipoise
